# A Novel Hybrid Method to Predict PM_2.5_ Concentration Based on the SWT-QPSO-LSTM Hybrid Model

**DOI:** 10.1155/2022/7207477

**Published:** 2022-08-16

**Authors:** Meng Du, Yixin Chen, Yang Liu, Hang Yin

**Affiliations:** ^1^Department of Finance, Shandong Technology and Business University, Yantai, Shandong 264005, China; ^2^Collaborative Innovation Center for Financial Service Transformation and Upgrading, Yantai 264005, China; ^3^School of Land Science and Technology, China University of Geosciences (Beijing), Beijing 100083, China; ^4^School of Economics and Management, Dalian University, Dalian 116622, China; ^5^Dalian Central Sub-Branch, The People's Bank of China, Dalian 116001, China

## Abstract

PM_2.5_ concentration is an important indicator to measure air quality. Its value is affected by meteorological factors and air pollutants, so it has the characteristics of nonlinearity, irregularity, and uncertainty. To accurately predict PM_2.5_ concentration, this paper proposes a hybrid prediction system based on the Synchrosqueezing Wavelet Transform (SWT) method, Quantum Particle Swarm Optimization (QPSO) algorithm, and Long Short-Term Memory (LSTM) model. First, the original data are denoised by the SWT method and taken as the input of the prediction model. Then, the main parameters of the LSTM model are optimized by global search based on the QPSO algorithm, which solves the problems of slow convergence and local extremum of traditional parameter training algorithms. Finally, the PM_2.5_ daily concentration data of Chengdu, Shijiazhuang, Shenyang, and Wuhan are predicted by the proposed SWT-QPSO-LSTM model, and the prediction results are compared with those of single prediction models and hybrid prediction models. The experimental results show that the proposed model achieves higher prediction precision and lower prediction error than other models.

## 1. Introduction

The problem of air pollution is increasingly significant as industrialization and urbanization progress, and it has attracted attention worldwide. PM_2.5_ (fine particles with aerodynamic diameter ≤2.5 *μ*m) mainly comes from the exhaust emission of urban traffic and the exhaust gas generated by various industrial activities. As the primary component of atmospheric pollution particles, PM_2.5_ is the culprit of haze weather. The epidemiological studies at home and abroad show that long-time exposure to a high concentration of PM_2.5_ will cause blood pressure rise, arrhythmia, and even stroke, myocardial infarction, atherosclerosis, and other diseases, seriously threatening human health [1]. Besides, PM_2.5_ also has adverse effects on climate change. It leads to abnormal rainfall, exacerbates the greenhouse effect, affects traffic visibility, and improves the traffic accident rate, thus hindering the development of the transportation industry and regional economic development [2]. Therefore, gathering PM_2.5_ concentration data in real time is critical for managing air pollution, preventing cardiovascular and cerebrovascular diseases, and improving regional climate, ecological balance, and regional economic development. Because of the serious negative impact of PM_2.5_ on people's life, more and more individuals are paying attention to PM_2.5_ concentration predictions. PM_2.5_ concentration prediction is considered to be an important and effective method to alleviate the negative impact of PM_2.5_.

Physical models and data-driven models are two types of PM_2.5_ concentration prediction methodologies. The physical model simulates the diffusion, propagation, and elimination process of PM_2.5_ through physical models. The advantage of this method is that it can illustrate the pollutant propagation and transformation process of PM_2.5_, which allows for better knowledge of PM_2.5_ pollution [3]. The typical physical models include the multiscale air quality model CMAQ developed by the U.S. Environmental Protection Agency (USEPA), the mesoscale weather prediction model chemical module WRF-Chem developed by the National Center for Atmospheric Research (NCAR) and the National Oceanic and Atmospheric Administration (NOAA), and the China Unified Atmospheric Chemical Environment (CUACE/Haze-fog) developed by the China Meteorological Administration. Because the diffusion mechanism of PM_2.5_ and other pollutants is very complex, the accuracy of the physical model is affected by many meteorological elements, such as temperature, humidity, wind direction, and season. Also, the physical model is often sensitive to the initial and boundary conditions and has high uncertainty. Besides, due to the time-consuming simulation process and the high calculation cost, the application of this model is greatly limited. In comparison, the data-driven model adopts statistical and machine learning methods to predict the PM_2.5_ concentration by mining the historical information and related influencing factors of PM_2.5_ concentration in meteorological observation stations. This model can obtain high-accuracy prediction results.

The data-driven air quality prediction models can be divided into three types: statistical prediction models, machine learning prediction models, and hybrid prediction models. The common statistical prediction models for PM_2.5_ concentration are ARIMA Model, grey prediction model (G, M), Markov model, and ANFIS [4–7]. However, the statistical prediction models are relatively simple and the prediction accuracy is relatively low. The prediction models based on machine learning mainly include Artificial Neural Network (ANN), Support Vector Machine (SVM), Support Vector Regression (SVR), and Least Squares Support Vector Machine (LS-SVM) [8–11]. However, SVM, SVR, and LS-SVM have high requirements for parameter selection and cannot deal with the problem of big data. ANN needs a lot of data for training and it is prone to overfitting in the process of data training and prediction.

The lack of a single appropriate method encourages the growth of hybrid models. To increase the model's prediction performance, a hybrid model integrates data preprocessing with various optimization techniques. The decomposition algorithm is commonly used in data preprocessing to reduce noise from the original data and extract the data's effective features. Through the decomposition algorithm, the original data can be transformed into a form suitable for machine learning model training and prediction. The decomposition algorithms used in PM_2.5_ concentration prediction mainly include empirical mode decomposition (EMD), variable mode decomposition (VMD), and wavelet decomposition [12–14]. EMD and VMD algorithms lack a mathematical basis, so they suffer from several problems such as wrong signals or pattern confusion. For the wavelet packet decomposition algorithm, there is no reliable and accurate expression of nonsmooth signals. An optimization algorithm is usually adopted to increase the accuracy of model prediction by optimizing the initial values and weights of machine learning models. Common optimization algorithms include the genetic algorithm, particle swarm optimization, and grey wolf algorithm [15]. Some studies combine the optimization algorithm with a machine learning prediction model to predict PM_2.5_ concentration. For example, Niu et al. [16] combined comprehensive ensemble empirical mode decomposition (CEEMD), SVR, and Grey Wolf Optimizer (GWO) to predict PM_2.5_ concentration. Sun and Sun [17] constructed a hybrid model that combines principal component analysis, LSSVM, and Cuckoo algorithm to predict PM_2.5_ concentration. Cheng et al. [18] believed that the prediction accuracy of hybrid models such as Wavelet-ANN, Wavelet-ARIMA, and Wavelet-SVM for PM2.5 is significantly higher than that of ARIMA, ANN, and SVM.

In recent years, the application of deep learning methods has achieved great success in numerous fields, such as image classification, natural language processing, and time series prediction. Some scholars have begun to use deep learning models to predict air quality, and the most successful prediction model is Long Short-Term Memory (LSTM). LSTM has the characteristics of long-term and short-term memory for time series data, so the prediction methods based on this model have good generalization ability and fault tolerance. Chang et al. [19] considered that the aggregated LSTM model achieves a better prediction effect for PM_2.5_ concentration than SVR, GBTR (Gradient Boosted Tree Regression), LSTM, and other models. Dhakal et al. [20] used Kathmandu Valley as a case study and discovered that the LSTM model predicted PM_2.5_ better than the SARIMA model. Some scholars construct hybrid models combining data preprocessing algorithms with LSTM models to predict PM_2.5_ concentrations. Yan et al. [21] proposed a hybrid model combining LSTM with the stationary wavelet transform, which is superior than SVR, LSTM, and convolutional neural network combining Long Short-Term Memory (CNN-LSTM). Zhang et al. [22] combined VMD and bidirectional LSTM (BiLSTM) to predict PM_2.5_ concentration, VMD algorithm is better than EMD algorithm in preprocessing, and, compared with SVR, MLP, and LSTM model, the prediction effect of BiLSTM has obvious advantages. Jin et al. [23] constructed a hybrid model that combines wavelet transform and nested LSTM to forecast PM_2.5_ concentration; the results show that the performance of the proposed method outperforms those of other models such as Decision Tree, Random Forest, MLP, EMD-LSTM, and VMD-LSTM. However, the above-mentioned papers do not use evolutionary algorithms to optimize the hyperparameters of the LSTM model, which may affect the prediction results of the models.

In this paper, a new hybrid model is developed to predict PM_2.5_ concentration in China using Synchrosqueezing Wavelet Transform (SWT), Long-Short Term Memory (LSTM), and Quantum Particle Swarm Optimization (QPSO). The contributions of this work are summarized as follows:

1. In data preprocessing, the SWT is used to decompose and reconstruct the data. As a classical time-frequency analysis method, the wavelet transform method has a solid mathematical theory basis, and it can accurately express the time-frequency local properties of signals. SWT is developed on the basis of wavelet transform theory; compared with other wavelet transforms, SWT reinstantiates energy only in the frequency direction and preserves the temporal resolution of the signal, which can improve the time-frequency resolution of the whole process without changing the clarity of the instantaneous points; these features of the SWT method are suitable for reconstructing PM_2.5_ data.
2. QPSO is applied to optimize the LSTM model. The selection of LSTM parameters affects the prediction accuracy of PM_2.5_. Based on the PSO algorithm and the principle of quantum mechanics, QPSO makes the motion state of particles follow random rules so that the optimization search range of particles can be expanded to the whole feasible solution space. Meanwhile, the global optimal solution can be found faster and more accurately. The experimental results indicate that the proposed hybrid prediction model may successfully improve the PM_2.5_ concentration prediction effect.

## 2. Methodology

### 2.1. Principle of Synchrosqueezing Wavelet Transform

Traditional wavelet analysis is widely used in signal denoising because of its good time-frequency characteristics and multiresolution characteristics. SWT is a time-frequency domain redistribution method proposed by Daubechie et al. [24], which is a further development of wavelet transform (WT). Based on the continuous wavelet transform (CWT), this method adds and recombines wavelet coefficients with the same instantaneous frequency; that is, the wavelet coefficients near the central frequency are squeezed to improve the fuzzy phenomenon in the scale direction. Meanwhile, the time-frequency curve is thinned so that there is no cross term, which can significantly improve the time-frequency resolution and reconstruct the signal without distortion. Using the SWT method, the energy in the time-scale plane is redistributed according to the module size of each element, and the plane is transformed into a time-frequency plane through special mapping. This method achieves good results in various applications, such as climate analysis, mechanical fault diagnosis, signal denoising, civil engineering structures, harmonic and interharmonic detection, and seismic signal extraction.

SWT is based on continuous wavelet transform (CWT), and the phase of the signal in the frequency domain processed by wavelet transform is not affected by scale transformation. CWT is applied to a signal sequence *s(t)*, and the expression of wavelet coefficient can be obtained as follows:

(1) $$\begin{array}{c} W_{s}\left(a,b;\phi \right)=\frac{1}{\sqrt{a}}\int_{-\infty }^{+\infty }s\left(t\right)\bar{\phi \left(\frac{t-b}{a}\right)}\text{d}t, \end{array}$$

where $\bar{\phi \left(t-b/a\right)}$ is the complex conjugate of the wavelet function; *a* is the scale parameter (also known as the stretching parameter) and *a* > 0; *b* ∈ *R* represents the translation parameter, and the wavelet basis function is

(2) $$\begin{array}{c} \phi_{a,b}\left(t\right)=\frac{1}{\sqrt{\left|a\right|}}\bar{\phi \left(\frac{t-b}{a}\right)}. \end{array}$$

According to equations (1) and (2), wavelet transform can be regarded as the convolution of wavelet function and signal after telescopic translation transform, and the convolution coefficients are arranged in the time-scale plane.

In practice, the collected signal sequence usually contains various noise and human factors. When the coefficient |*W*|_*s*_ obtained by wavelet transform is close to 0, the phase of *W*_*s*_ will be relatively unstable. For a threshold *γ*, if |*W*_*s*_| ≤ *γ*, this part is filtered out. According to different noise levels of wavelet coefficients, the threshold of each layer of wavelet coefficients can be adaptively obtained by using the following equation:

(3) $$\begin{array}{c} \gamma =\sigma_{\eta }\sqrt{2\mathrm{lg}\;n},\\ \sigma_{\eta }=\frac{\text{median}\left\{\left||W_{s}\left(a_{1:n_{v}},b\right)-\text{median}\left[W_{s}\left(a_{1:n_{v}},b\right)\right]\right|\right\}}{0.6745}, \end{array}$$

where *n* represents the number of sampling points of the original signal; *σ*_*η*_^2^ is the noise variance; and *W*_*s*_(*a*_1:*n*_*v*__, *b*) is the wavelet coefficient on the length of the scale factor.

In fact, the obtained wavelet coefficients spectrum always diffuses in the scale direction, and the focusing performance is unsatisfactory. This makes the time-frequency diagram blurred. However, the phase of wavelet coefficients is not affected by scale transformation, so the instantaneous frequency *W*_*s*_(*a*, *b*) can be calculated according to wavelet coefficients *W*_*s*_(*a*, *b*; *ϕ*) (|*W*_*s*_| > *γ*):

(4) $$\begin{array}{c} W_{s}\left(a,b\right)=\frac{-i}{2\pi W_{s}\left(a,b;\phi \right)}\frac{\partial W_{s}\left(a,b;\phi \right)}{\partial b}. \end{array}$$

Through the calculation of equation (4), the time-frequency domain redistribution is realized, and the time-scale plane is transformed into a time-frequency plane. This is the basic idea of compressed wavelet transform. Based on this, the value of the neighborhood interval [*ω*_*l*_ − 1/2Δ*ω*, *ω*_*l*_+1/2Δ*ω*] of any frequency *ω*_*l*_ can be compressed to *ω*_*l*_. The formula of synchronous compression transformation can be expressed as

(5) $$\begin{array}{c} T_{S}\left(\omega_{l},b\right)={\left(\Delta \omega \right)}^{-1}\sum_{a_{k}：\left|\omega_{s}\left(a,b\right)-\omega l\leq △\omega /2\right|}W_{s}\left(a,b;\phi \right)a_{k}^{-3/2}\Delta a_{k}, \end{array}$$

where *a*_*k*_ is the discrete scale and *k* is the number of scales. When the signal is in the discrete state, the scale coordinate Δ*a*_*k*_ and frequency coordinates Δ*ω* are discrete values:

(6) $$\begin{array}{c} \Delta a_{k}=a_{k}-a_{k-1},\\ \Delta \omega =\omega_{l}-\omega_{l-1}. \end{array}$$

SWT is a time-frequency rearrangement algorithm. Different from other rearrangement algorithms in the past, SWT can not only increase the time-frequency resolution ratio but also reconstruct the characteristic segment of the signals in the time-frequency diagram. Thus, SWT is reversible, and its inverse transform (ISWT) can be denoted as

(7) st=2ReCφ−1∑lTSωl,bΔω,

(8) $$\begin{array}{c} C_{\varphi }=\int_{0}^{\infty }\frac{\bar{\psi^{*}\left(\xi \right)}}{\xi }\text{d}\xi . \end{array}$$

Signal reconstruction can be realized by equation (7), where $\bar{\psi^{*}\left(\xi \right)}$ represents the conjugate Fourier transform of a basic wavelet function equation.

To sum up, the extraction of signal sequences based on SWT consists of the three following steps:

1. Use CWT to transform signal *s(t)* to obtain the wavelet coefficients *W*_*s*_(*a*, *b*; *ϕ*)
2. Obtain the instantaneous frequency from the wavelet coefficients (|*W*_*s*_| > *γ*) and obtain the synchronous compression coefficient *T*_*S*_(*ω*_*l*_, *b*) by synchronous compression
3. Use the effective signal for synchronous compression transformation and reconstruction to realize signal extraction

### 2.2. Principle of Long Short-Term Memory Neural Network

Recurrent neural network (RNN) is often used to handle the data with sequence changes. RNN is a network containing loops, which allows information persistence. Every neural network module of RNN transmits the message to the next module through replication. However, when the interval between the predicted position and relevant information magnifies to a certain extent, RNN will lose its learning ability because it cannot connect such far information. LSTM is a specific RNN proposed by Hochreiter and Schmidhuber [25], which can solve the problem of gradient disappearance or explosion in long sequence training. LSTM achieves better performances on training longer sequences than conventional RNN.

Unlike the standard RNN with only one transmission state (*h*^*t*^) and a single tanh layer, LSTM has two transmission states (*c*^*t*^ and *h*^*t*^), and its duplicating module includes four layers that interact in a special way. Besides, LSTM adds a “processor” named cell to the algorithm, which can estimate whether the input information is serviceable. There are three gates in a cell: input gate, forgetting gate, and output gate. These gates protect and control the state of the cells. When information enters the LSTM network, the cell determines whether this information is useful according to the rules. Meanwhile, the information consistent with the algorithm identification is left, and the inconsistent information is forgotten through the forgetting gate.

The processing process of LSTM is mainly divided into three steps. The first step discards the irregular information in the cell state through the forgetting gate according to the rules. The gate reads *h*_*t* − 1_ and *x*_*t*_ and assigns each cell a value between 0 and 1 in the cell state *C*_*t* − 1_, where 1 indicates “completely reserved” and 0 indicates “completely discarded.” At this time, the cell status output is

(9) $$\begin{array}{c} f_{t}=\sigma \left(W_{f}\cdot \left[h_{t-1},x_{t}\right]+b_{f}\right). \end{array}$$

The second step determines the new information stored in the cell state according to the rules. The Sigmoid layer is called “input layer,” and it resolves the value that needs to be updated. The tanh layer creates a new candidate value vector, and $\tilde{C_{t}}$ is involved in the conditions. The formula for input door is as follows:

(10) $$\begin{array}{c} i_{t}=\sigma \left(W_{i}\cdot \left[h_{t-1},x_{t}\right]+b_{i}\right),\\ \tilde{C_{t}}=\tanh\left(W_{c}\cdot \left[h_{t-1},x_{t}\right]+b_{C}\right). \end{array}$$

The state of an old cell is updated so that *C*_*t* − 1_ is updated to *C*_*t*_. The expression is as follows:

(11) $$\begin{array}{c} C_{t}=f_{t}*C_{t-1}+i_{t}*\tilde{C_{t}}. \end{array}$$

The third step decides the final output value based on the state of the cells. The output gate determines the information output by the cells, and its formula is as follows:

(12) $$\begin{array}{c} o_{t}=\;\sigma \left(W_{o}\cdot \left[h_{t-1},x_{t}\right]+o\right),\\ h_{t}=o_{t}*\;\tanh\left(C_{t}\right). \end{array}$$

Here, *W* is the weight measurement of every door, and *b* is the deviation.

### 2.3. Principle of Quantum Particle Swarm Optimization

QPSO is an improved PSO algorithm proposed by Sun et al. [26] to handle the problems of premature convergence and local extremum of standard PSO algorithm.

In the PSO algorithm, particles have two attributes: location (*X*) and velocity (*V*). In a *D*-dimensional solution space, the state of particle *i* is expressed as *X*_*i*_=(*x*_*i*1_, *x*_*i*2_,…, *x*_*iD*_) and *V*_*i*_=(*v*_*i*1_, *v*_*i*2_,…, *v*_*iD*_). The fitness function is calculated to determine the fitness to express the advantages and disadvantages of the particle. Each time the particle is updated, its fitness needs to be recalculated. The particle with the best fitness is selected to update the optimal positions of individuals *P*_best_=(*p*_*i*1_, *p*_*i*2_,…, *p*_*iD*_) and the group optimal position *p*_*g*best_=(*P*_*g*1_, *P*_*g*2_,…, *P*_*gD*_). The update of the speed and position of particles at a certain time depends on the state of particles at the previous time. Thus, the speed and position of particles at any time are not random, and the particle optimization search cannot cover the whole solution space and falls into the local extremum easily. That is, the PSO algorithm is not a global convergence algorithm, and the final optimal solution obtained by this algorithm is the local optimal solution instead of the global optimal solution. With the expansion of applications, the PSO algorithm suffers from some problems, such as early convergence and dimension explosion.

Following the principle of quantum mechanics, QPSO algorithm uses the quantum behavior of particles for optimization. Specifically, the particles follow the random rule of quantum motion. The motion state of the particles at present is no longer affected by that of the previous moment. Thus, the particle search scope can be extended to the whole feasible solution space, and the global optimum solution can be obtained faster and better. QPSO algorithm improves the iterative behavior and optimization strategy of particle swarm optimization, and it has the advantages of fewer parameters, high efficiency, and global optimization.

When a particle following the quantum motion rules moves, its velocity and position are uncertain. In this case, the motion state of the particle is expressed by the probability density function (PDF) of the particle at a certain point in the solution space. In our study, PDF is replaced by the square of the wave function, and the exact position of the particle is simulated by the Monte Carlo method. The update formula of particle position is

(13) $$\begin{array}{c} x_{i}^{t+1}=\left\{\begin{array}{c} P_{i}^{t}+\beta *\left|m_{\text{best}}-x_{i}^{t}\right|*\;\ln\left(\frac{1}{\mu }|,\right),\;\mu >0.5\\ P_{i}^{t}-\beta *\left|m_{\text{best}}-x_{i}^{t}\right|*\;\ln\left(\frac{1}{\mu }\right),\;\mu \leq 0.5 \end{array},\right. \end{array}$$

where *P*_*i*_^*t*^=(*P*_*i*1_^*t*^, *P*_*i*2_^*t*^,…, *P*_*iN*_^*t*^) is the random point where the particle moves in the feasible space; *β* is the contraction expansion control coefficient; *μ* is identified as a stochastic number within the range of [0, 1]; *m*_best_ represents the average value of the historical optimal position of particles, and its computing formula is as follows:

(14) $$\begin{array}{c} m_{\text{best}}=\frac{1}{N}\sum_{i=1}^{N}P_{\text{best}\_i}^{t}=\left(\frac{1}{N}\sum_{i=1}^{N}P_{{\text{best}}_{i},1}^{t},\frac{1}{N}\sum_{i=1}^{N}P_{{\text{best}}_{i},2}^{t},\ldots ,\frac{1}{N}\sum_{i=1}^{N}P_{{\text{best}}_{i},D}^{t}\right), \end{array}$$

where *N* is the total number of particles in the population; *D* is the particles' dimension; *P*_best_*i*_^*t*^ is the individual optimal position of the *i*-th particle in the current number of iterations. The optimal placement of individual particles interacts with the optimal location of the global particle swarm during particle motion, and *P*_*i*_ is constantly updated. The iterative equation of the *i*-th particle is

(15) $$\begin{array}{c} P_{i}^{t}=\varphi P_{\text{best}\_i}^{t}+\left(1-\varphi \right)p_{g\text{best}}^{t}, \end{array}$$

where *p*_*g*best_^*t*^ is the historical optimal location of the group and *φ* is a random number with [0, 1]. The procedure of the QPSO algorithm is illustrated in Algorithm 1.

## 3. The Hybrid Model of PM_2.5_ Concentration Prediction

### 3.1. Structure of the Prediction Model

**Figure 1:**
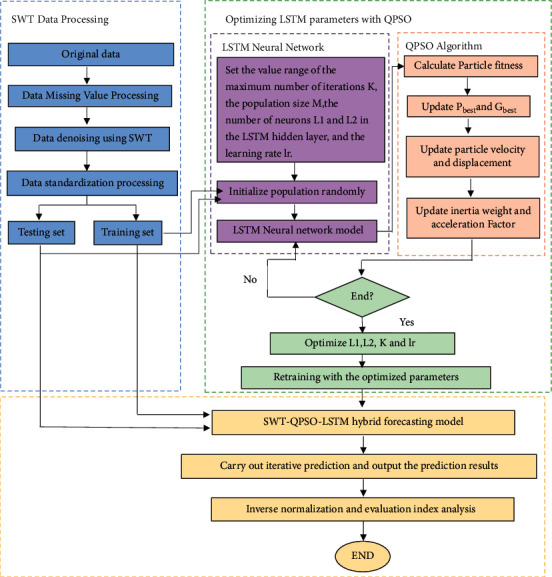
Structure of the PM_2.5_ concentration prediction model.

The PM_2.5_ concentration prediction model studied in this paper mainly consists of the four following components:

- *Data Selection*. PM_2.5_ is the main component of smog particles. Exposed to high concentrations of PM_2.5_ for a long time, people will suffer from an increased risk of respiratory, cardiovascular, and cerebrovascular diseases and even cancer. Therefore, the PM_2.5_ daily concentration data of a certain area is taken as the experimental data in this paper.
- *Data Preprocessing*. There are some problems in the collected data, such as missing values, anomalies, and inconsistent dimensions. Therefore, missing value processing and standardization need to be performed on the experimental data before simulation analysis. In the experiment, the SWT method is used for data preprocessing.
- *Neural Network*. The traditional neural network falls into the local extremum easily. Also, its convergence speed is slow, and the prediction precision needs to be improved. This paper uses QPSO to optimize the parameters of the LSTM neural network to construct a PM_2.5_ concentration prediction model.
- *Analysis of Prediction Results*. In this paper, the prediction precision of the simulation experiment is analyzed by calculating the values of RMSE, MAE, and MAPE. The structure of the prediction model is illustrated in Figure 1.

### 3.2. SWT-QPSO-LSTM Hybrid Prediction Model

The key parameters in the SWT-QPSO-LSTM hybrid prediction model include the number of neurons in LSTM (*L*1 and *L*2), learning rate (*ε*), and training iterations (*k*). The four key parameters are taken as the characteristics of particle optimization, and the LSTM model is adjusted and optimized by the QPSO algorithm. The overall process of the algorithm design and optimization is as follows:

(a) Standardizing the PM_2.5_ historical concentration data processed by the SWT method. Because the LSTM neural network model is sensitive to the scale of input data, a large data scale will affect the training effect of the model. The daily PM_2.5_ concentration data of 36 months before the sample data is taken as the training set, and the PM_2.5_ concentration data of 12 months after the sample data is taken as the testing set. The data can be standardized by the following equation:
  (16) $$\begin{array}{c} y=\left(x-x\text{mean}\right)*\left(\frac{y\text{std}}{x\text{std}}\right)+y\text{mean,} \end{array}$$
where the default value of *y*mean is 0 and the default value of *y*std is 1; *x* is the original PM_2.5_ concentration data; *x*mean is the mean value of the original data; *x*std is the standard deviation of the original data, and *y* is the standardized PM_2.5_ concentration data with a mean value of 0 and standard deviation of 1.
(b) Initializing the particle swarm parameters, including the population number, the learning rate, the maximum number of iterations, and the value range of particle position and velocity.
(c) According to experience, this paper selects the initial values of *L*1, *L*2, *ε*, and *k* to establish the LSTM model. Then, the model is trained on the training set, and the results are compared with those obtained on the testing set. Finally, a set of superparameters are obtained by searching to minimize the prediction error of LSTM. This paper takes the mean square error (MSE) as the fitness function, which is calculated as follows:
  (17) $$\begin{array}{c} \text{MSE}=\frac{1}{M}\overset{M}{\underset{m=1}{\sum }}{\left(y_{m}-\hat{y_{m}}\right)}^{2}. \end{array}$$
In the previous formula, *y*_*m*_ and $\hat{y_{m}}$, respectively, represent the real value and predicted value of the sample.
(d) The global optimal position gbest and local optimal position pbest are determined by the initial fitness value of the particles, and they are regarded as the historical optimal positions. The speeds and positions of the particles are updated. Then, the corresponding particle fitness value is calculated and compared with the local and global optimal solution to improve the accuracy.
(e) Judging whether the termination condition of the algorithm is satisfied (the fitness value of the particles tends to be stable or the maximum number of iterations is reached). If the termination condition is satisfied, the optimal parameter values of the SWT-QPSO-LSTM model are obtained; otherwise, go to (d).
(f) The prediction results on the test set are obtained by the SWT-QPSO-LSTM prediction model constructed with the optimal parameter values. The prediction results are analyzed and summarized according to the evaluation indexes.

### 3.3. Model Evaluation Indexes

In this study, the indexes of root mean square error (RMSE), mean absolute error (MAE), and mean absolute percentage error (MAPE) are used to evaluate the prediction performance. The lower the values of RMSE, MAE, and MAPE, the better the prediction results of the proposed model. The calculation formulas of these evaluation indexes are as follows:

(18) $$\begin{array}{c} \text{RMSE}\left(y_{i},\hat{y_{i}}\right)=\sqrt{\sum_{i=1}^{n}y_{i}-{\hat{y_{i}}}^{2}/n},\\ \text{MAE}=\frac{1}{N}\sum_{i=1}^{N}\left|y_{i}-\hat{y_{i}}\right|,\\ \text{MAPE}\left(y_{i},\hat{y_{i}}\right)=\frac{1}{n}\sum_{i=1}^{n}\left|\frac{y_{i}-\hat{y_{i}}}{y_{i}}\right|*100\%, \end{array}$$

where *n* is the number of test samples; *y*_*i*_ and $\hat{y_{i}}$ are, respectively, the truth value and predicted value at time *i*.

## 4. Simulation Analysis

### 4.1. Data Selection and Analysis

In order to verify the predictive validity of our constructed model, four Chinese cities with different regions and different air quality levels, Chengdu, Wuhan, Shenyang, and Shijiazhuang, are selected as samples for empirical study in this paper. Chengdu, the largest city in southwest China, has a more reasonable industrial structure and better air quality, and its air quality index is in the top of the 168 key cities in China. Wuhan, an important city in central China, has seen significant improvement in its air quality with the transformation of its industrial structure; now the Air Quality Index of Wuhan is ranked in the upper middle of China's 168 key cities. Shenyang is an important heavy industrial city, and its long-term economic development approach has led to rising resource and energy consumption; the air pollution problem is becoming increasingly acute; the Air Quality Index of Shenyang is in the lower reaches of 168 key cities in China. Shijiazhuang is located in the core of the Beijing-Tianjin-Hebei city cluster, it has poor air quality due to its special geographical location and unreasonable industrial layout, and its air quality index is at the end of 168 key cities in China. If the constructed model can well predict the air quality for these four cities, it means that the constructed model is effective. This paper uses the daily PM_2.5_ concentration data from January 1, 2018, to March 31, 2021, as the case study data to determine the effectiveness of the constructed model. The data set can be obtained from the public air quality query website (https://www.aqistudy.cn/historydata/). The PM_2.5_ concentration unit is *μ*g/m^3^, and there are 1,186 data points. The analysis revealed the phenomenon of missing values in the data, so the interpolation method was used to anomalously process the raw data, the data is divided into training and test sets according to a ratio of 7.3, the training data are used to fit the model, the test data are used to evaluate the performance of the constructed model, and their descriptive statistics are shown in Table 1.

### 4.2. Experimental Process

The section is divided into two parts: data preprocessing and hybrid model prediction.

#### 4.2.1. Data Preprocessing Results after SWT

**Figure 2:**
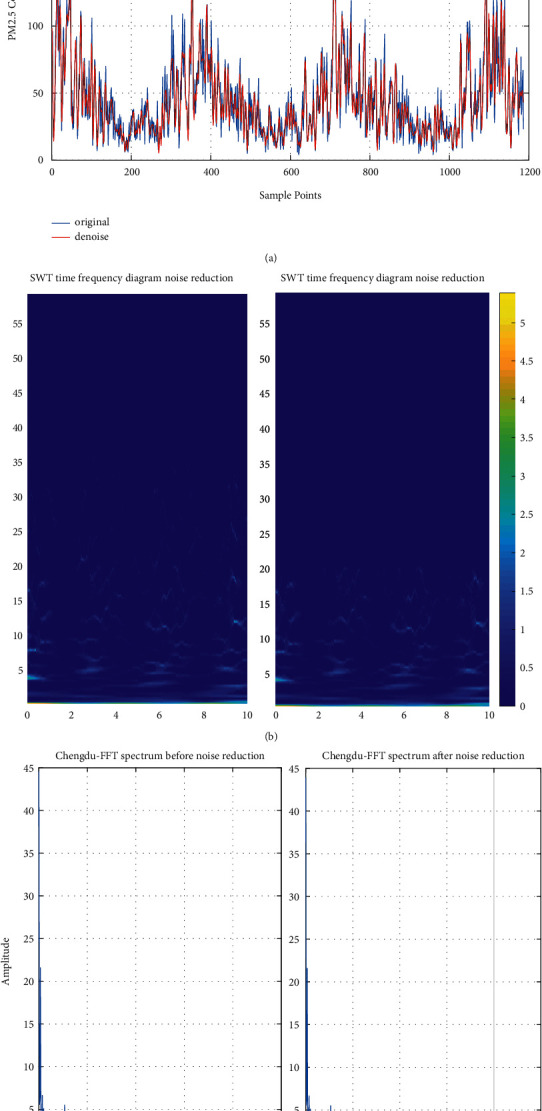
SWT results of the raw PM_2.5_ concentration data of Chengdu.

In this section, SWT is used to reduce the noise of the PM_2.5_ concentration data.  Figure 2 shows the SWT results of the original data, and Figure 2(a) presents the PM_2.5_ concentration data before and after denoising; Figure 2(b) presents the SWT time-frequency diagram of the original data before and after noise reduction; Figure 2(c) presents the FFT spectrum of the original data before and after noise reduction. It can be seen from Figures 2(b) and 2(c) that the noise at the low frequency of SWT is well suppressed, and the frequency component is compressed. This indicates that SWT can suppress random noise and extract characteristic frequency. Based on this, the noise interference of the original sample data is reduced by the SWT method to achieve more accurate prediction results.

#### 4.2.2. QPSO-LSTM Prediction Model Based on the SWT Method

To increase the prediction precision of the LSTM model, the QPSO algorithm is adopted to optimize the parameters of the LSTM model established on the SWT data preprocessing method. As we need to optimize the 4 parameters of the LSTM model, the optimization dimension is set to 4, in order to ensure that the parameter optimization could converge, the number of populations is set as large as possible, the population number is set to 100, and the value range of the learning rate is determined as [0.001, 0.01].

**Figure 3:**
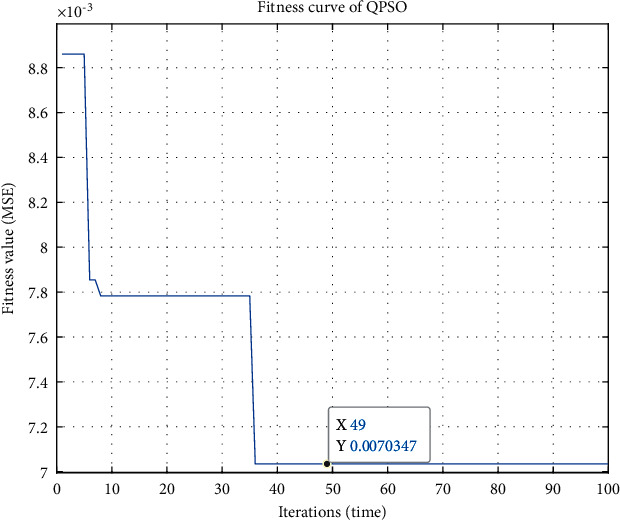
The variation of the particle fitness of the SWT-QPSO-LSTM model.

Taking the PM_2.5_ concentration data of Chengdu as an example, the variation of the particle fitness with the increasing iterations of the SWT-QPSO-LSTM model is shown in Figure 3. The particle fitness finally stabilizes at 0.0070347.

**Figure 4:**
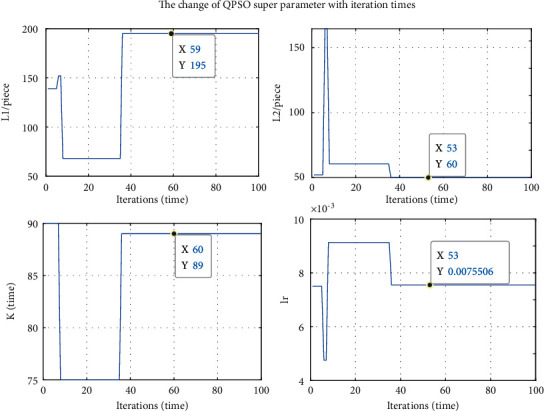
Variation of parameters in the QPSO-LSTM model based on SWT.

The variation of the maximum number of optimization iterations (*K*), learning rate (*ε*), and the number of hidden layer neurons (*L*1 and *L*2) with the number of model iterations is shown in Figure 4. It can be found that all four parameters converge at a certain point; the final parameter optimization result of the SWT-QPSO-LSTM prediction model is *K* = 89, *ε*  = 0.0075506, *L*1 = 195, and *L*2 = 60. The results of parameter optimization for other models are shown in Table 2.

**Figure 5:**
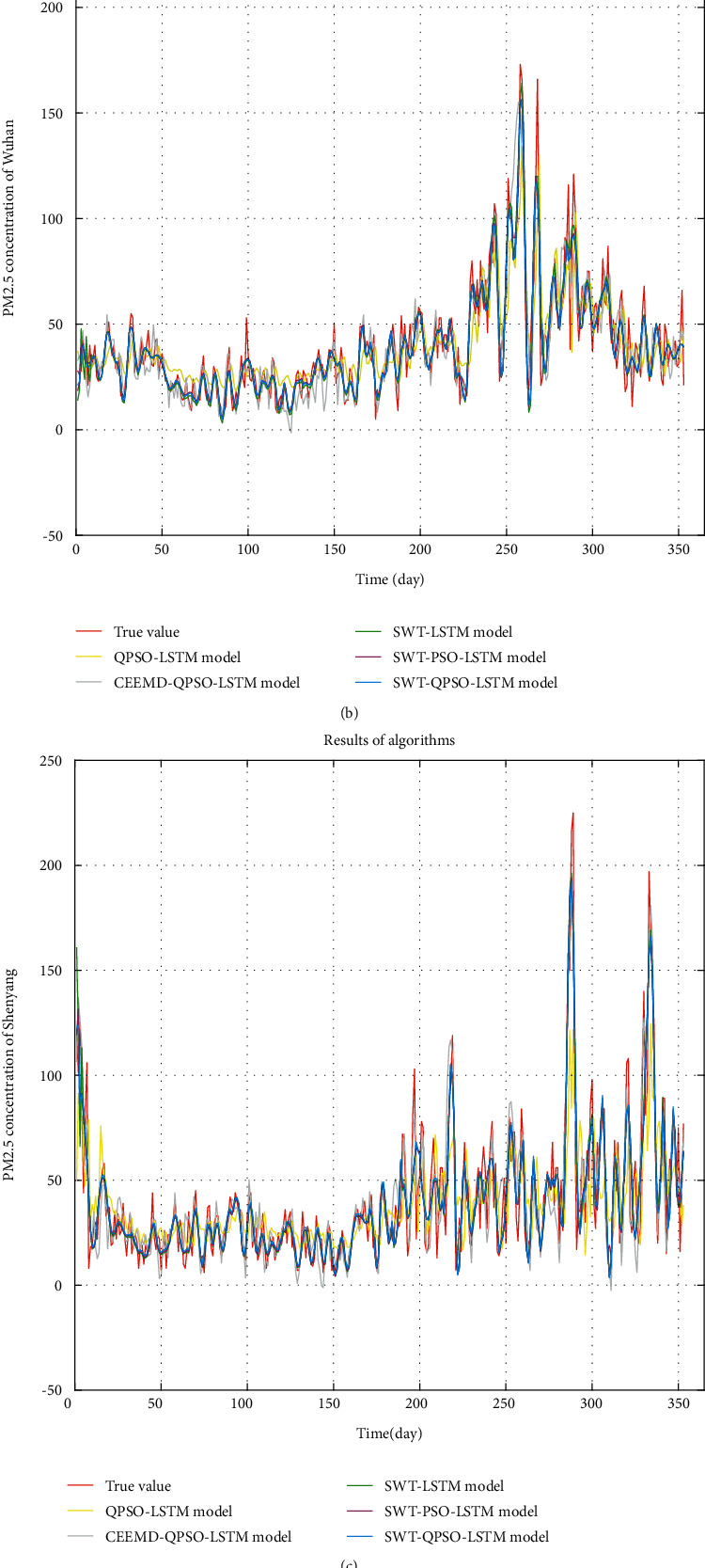
The performance of five prediction models on the PM_2.5_ concentration data of Chengdu, Wuhan, Shenyang, and Shijiazhuang.

To verify the effectiveness of the SWT-QPSO-LSTM hybrid model proposed in this paper, the hybrid model is compared with the single prediction model SWT-LSTM and the hybrid prediction model SWT-PSO-LSTM. Besides, to prove that the accuracy of the PM_2.5_ prediction model can be better improved by the SWT data processing method, the hybrid model proposed in this paper is compared with the QPSO-LSTM model without SWT processing and the QPSO-LSTM prediction model with the CEEMD data processing method. CEEMD is a decomposed method that is widely used in processing time-series data.  Table 3 shows the evaluation indexes of the five prediction models on the PM2.5 concentration data of Chengdu, Wuhan, Shenyang, and Shijiazhuang. Figures 5(a)–5(d) show the prediction results obtained by five prediction models (SWT-LSTM, SWT-PSO-LSTM, SWT-QPSO-LSTM, CEEMD-QPSO-LSTM, and QPSO-LSTM) on the PM_2.5_ concentration data of Chengdu, Wuhan, Shenyang, and Shijiazhuang, respectively.

Data preprocessing has a greater impact on the prediction results, and the prediction results of the models with data preprocessing using either the SWT or CEEMD methods are significantly better than the prediction results of the models without data preprocessing; compared to the CEEMD method, data preprocessing by SWT method results have smaller prediction errors. The LSTM models, whose parameters are optimized by the swarm intelligence algorithm, have the better prediction; among them, the LSTM model optimized by QPSO algorithm predicts slightly better than those optimized by PSO. The above analysis results indicate that the SWT-QPSO-LSTM hybrid prediction model proposed in this paper achieves less prediction error than other prediction models. This verifies the feasibility and effectiveness of the established model.

## 5. Conclusion

Aiming at the prediction of PM_2.5_ concentration, this paper proposes a hybrid prediction model based on the LSTM neural network and the QPSO optimization algorithm. The data used in this study is preprocessed by the SWT method, and the obtained data is input into the QPSO-LSTM model for simulation and prediction. The prediction results of the proposed model are analyzed, and the model is compared with other hybrid models. It is proved that the proposed method achieves better feasibility and effectiveness than other methods. The specific conclusions are as follows:

1. The use of the SWT method to preprocess nonstationary signals can realize signal reconstruction, which improves the accuracy of subsequent data prediction
2. The use of the QPSO optimization algorithm to optimize the key parameters of the LSTM model can effectively avoid the impact of parameter setting on the prediction performance
3. The proposed SWT-QPSO-LSTM hybrid prediction model achieves better prediction performance than QPSO-LSTM, CEEMD-QPSO-LSTM, SWT-LSTM, and SWT-PSO-LSTM, indicating that the SWT-QPSO-LSTM model can effectively predict PM_2.5_ concentration

The model proposed in this paper provides a theoretical basis for air pollution control; however, there are still some limitations. How to balance prediction accuracy and complexity of the model has always been a difficult task. Our proposed model has obvious advantages in prediction accuracy; however, the time spent on training the deep model is also worthy of attention. In the future, we consider embedding the model in the parallel framework such as Apache Spark to improve the model running efficiency. In addition, meteorological factors are not considered in this paper; we will add more influencing factors and use spatiotemporal deep learning neural network model to improve PM_2.5_ prediction accuracy in the future work.

## Figures and Tables

**Algorithm 1:**
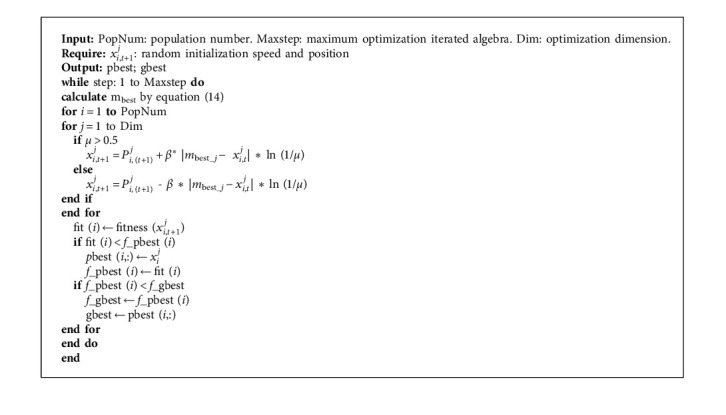
QPSO algorithm.

**Table 1:** Descriptive statistics of the PM_2.5_ concentration data.

Data set	Min	Max	Mean	Std	Kurtosis

Chengdu	4	201	36.524	28.520	2.308
Wuhan	5	202	36.866	27.1388	4.203
Shenyang	6	265	33.954	33.155	6.418
Shijiazhuang	9	354	50.253	51.1982	51.198

**Table 2:** Parameter optimization results by evolutionary algorithm.

	Hybrid model	*K*	*ε*	*L*1	*L*2

Chengdu	SWT-QPSO-LSTM	89	0.0075506	195	60
	SWT-PSO-LSTM	83	0.004508	171	138
	CEEMD-QPSO-LSTM	60	0.008519	131	184
	QPSO-LSTM	25	0.009833	115	93

Shenyang	SWT-QPSO-LSTM	99	0.0039368	110	146
	SWT-PSO-LSTM	99	0.003524	162	166
	CEEMD-QPSO-LSTM	94	0.0031287	96	168
	QPSO-LSTM	27	0.005712	138	172

Shijiazhuang	SWT-QPSO-LSTM	60	0.0070699	195	67
	SWT-PSO-LSTM	87	0.0052502	139	74
	CEEMD-QPSO-LSTM	81	0.0029861	142	122
	QPSO-LSTM	24	0.0098442	152	57

Wuhan	SWT-QPSO-LSTM	83	0.0064491	102	98
	SWT-PSO-LSTM	78	0.0063656	169	160
	CEEMD-QPSO-LSTM	37	0.0021851	118	123
	QPSO-LSTM	17	0.0068847	155	138

**Table 3:** The evaluation indexes of the results obtained by the five models.

City	Prediction model	Evaluating indicator		
		RMSE	MAE	MAPE

Chengdu	SWT-QPSO-LSTM	2.2225	1.4693	3.9421%
	SWT-PSO-LSTM	2.1131	1.4601	3.9672%
	SWT-LSTM	3.507	2.3277	6.9107%
	CEEMD-QPSO-LSTM	14.3974	10.6858	34.9991%
	QPSO-LSTM	18.005	12.9346	43.1974%

Wuhan	SWT-QPSO-LSTM	1.8506	1.1903	3.465%
	SWT-PSO-LSTM	1.7679	1.1931	3.6633%
	SWT-LSTM	2.6409	1.5212	4.5527%
	CEEMD-QPSO-LSTM	11.9843	8.7563	26.729%
	QPSO-LSTM	15.4004	11.2529	39.6277%

Shenyang	SWT-QPSO-LSTM	3.1317	1.9897	5.2223%
	SWT-PSO-LSTM	3.156	1.9687	5.3339%
	SWT-LSTM	4.5356	2.2683	6.0506%
	CEEMD-QPSO-LSTM	14.2067	10.5664	37.9651%
	QPSO-LSTM	23.4806	16.5365	58.4215%

Shijiazhuang	SWT-QPSO-LSTM	3.0535	2.1581	5.3658%
	SWT-PSO-LSTM	3.2271	2.3048	5.6412%
	SWT-LSTM	4.6335	3.695	9.4836%
	CEEMD-QPSO-LSTM	18.3614	13.9762	34.3598%
	QPSO-LSTM	26.3039	19.4663	46.8304%

## Data Availability

The data used to support the findings of this study are available from the corresponding author upon request.
